# A Protracted Course of Herpes Simplex Virus Type 1 Encephalitis With Persistent Cerebrospinal Fluid Polymerase Chain Reaction Positivity Post Treatment

**DOI:** 10.7759/cureus.18107

**Published:** 2021-09-19

**Authors:** Andrew Abbott, Heather Lusby, Shehla P Islam

**Affiliations:** 1 Department of Infectious Diseases and Global Medicine, University of Florida College of Medicine, Gainesville, USA

**Keywords:** hsv-1, encephalitis, acyclovir, persistent hsv in csf, protracted hsv course

## Abstract

Herpes simplex virus type 1 encephalitis presenting as an undulating course for more than two weeks prior to treatment. Despite 21 days of intravenous acyclovir, the virus remained detectable in the cerebrospinal fluid. The patient was treated with an additional 21 days of acyclovir with further improvement in mental status.

## Introduction

Herpes simplex virus type 1 (HSV-1) is a double stranded DNA virus that is the most common infectious cause of sporadic encephalitis [1,2]. Worldwide, 60-90% of older adults are estimated to be seropositive for HSV-1 [1]. However, the yearly prevalence of encephalitis is only 0.2-0.4 in 100,000, with the majority of cases in adults over 50 years of age [1,2]. HSV-1 initially enters the body via mucosal membranes and breaks in the skin of a susceptible individual. After initial infection, the virus can enter the central nervous system leading to a rapidly progressing encephalitis that continues to worsen unless treated [1,3]. Untreated, mortality is high; estimated at around 70% [4]. The mainstay of treatment is intravenous acyclovir for 14-21 days. We describe a case of HSV-1 encephalitis that presented with a protracted course prior to diagnosis and persistence of detectable HSV-1 DNA in the cerebrospinal fluid (CSF) despite appropriate treatment.

## Case presentation

A 67-year-old veteran with a history of seizures, currently on levetiracetam and valproic acid, chronic kidney disease stage III, and bilateral below the knee amputations, presented to the ED due to bleeding from the site of his recent right amputation after mild trauma. The patient lived at home, alone, and had no electricity or running water. He was admitted for further supportive care, which included wound care and social support for a smooth transition to short-term rehabilitation.

The patient’s hospitalization was uneventful until day 10 of admission when he developed a fever of 38.3⁰C and was started on ceftriaxone with concern for a possibly infected tooth and further workup was initiated. However, despite antimicrobial therapy, his fever increased to a maximum of 39.4⁰C and he became hypotensive with an elevated white blood cell count of 16,200 cells/mm3 with a neutrophilic predominance and was minimally responsive. Lactic acid was 1.4 mmol/L, erythrocyte sedimentation rate 63 mm/hr, and procalcitonin 0.16 ng/mL. His levetiracetam level was 24.8 mcg/mL and valproic acid level was 43.6 ug/mL. His antimicrobials were broadened to vancomycin and cefepime and he was transferred to the medical intensive care unit. He was treated aggressively with IV fluids with an appropriate response in his blood pressure. His mental status improved but he was noted to still have some mild confusion with orientation questions. The workup was remarkable for greater than 180 white blood cells (WBC) and red blood cells (RBC) in the urine. Urine cultures only grew yeast and blood cultures were negative but he had received antibiotics prior to urine collection. At that time, the source of his sepsis was thought to be urinary.

**Figure 1:**
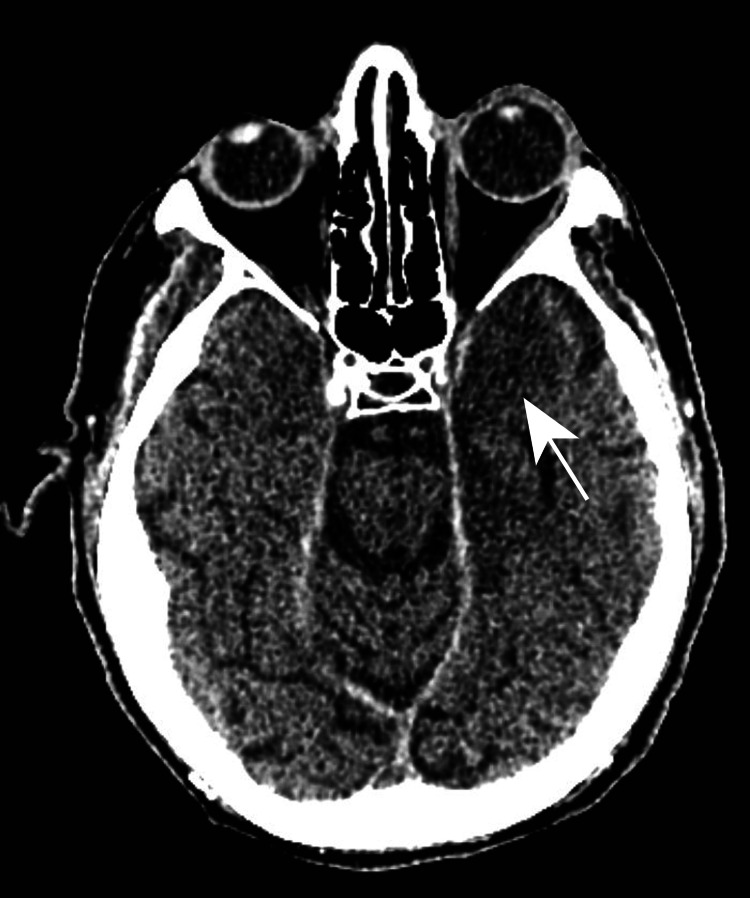
CT scan of the brain with hypodensity in the left anterior temporal pole

Despite broad-spectrum antibiotics, he continued to have a fever. He had difficulty with the pronunciation of words, was not following commands, and could move all extremities spontaneously. He was evaluated for non-infectious causes of febrile illness with concerns for drug fever due to his antiepileptics. Neurology was also following, and both antiepileptics were stopped. Vancomycin was discontinued after two days and cefepime was discontinued after five days as no other bacterial source of infection was isolated; cultures remained negative and he had continued to have fevers despite this therapy. On the day of cefepime cessation, six days after fever onset, he underwent his first CT scan that showed a new hypodensity in the left anterior temporal pole with concern for edema (Figure 1).

Initially, the focal lesion was thought not to be the cause of his fevers or encephalopathy as his symptoms were considered more of a global phenomenon. The patient again had fevers of up to 38.9⁰C and had increasing oxygen requirement. Chest x-ray was concerning for right middle/lower lobe pneumonia and urine culture grew yeast so the patient was restarted on cefepime and vancomycin with the addition of fluconazole. Speech therapy evaluation revealed evidence of aspiration and patient defervesced on antibiotic therapy.

**Figure 2:**
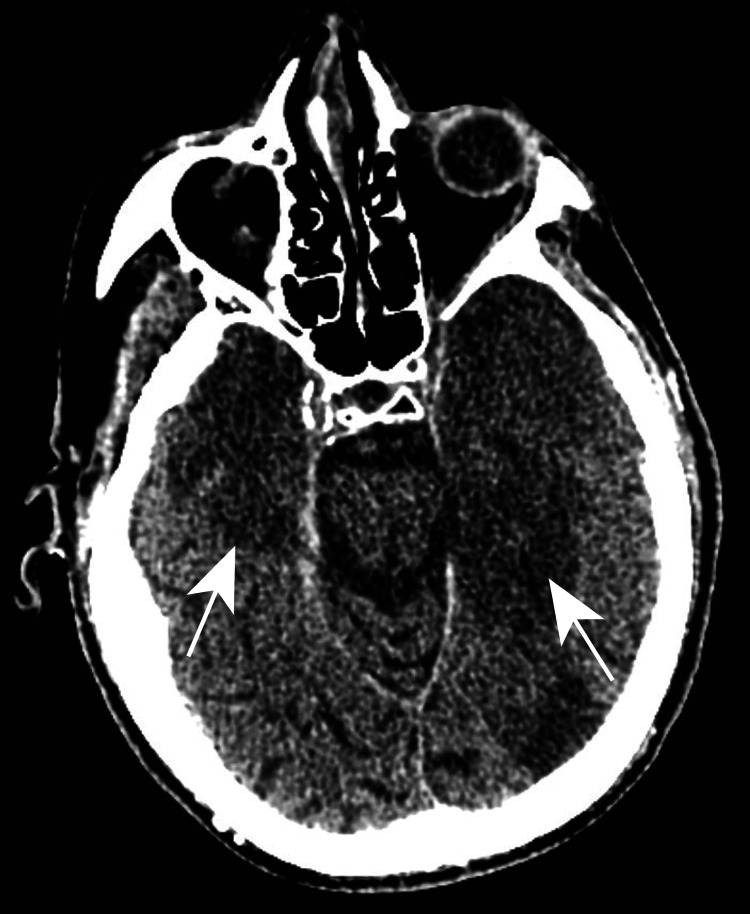
CT of the head with worsening hypoattenuation in the left temporal lobe and new right temporal lobe hypoattenuation

Mental status was notably still poor, which lead to aspiration followed by worsening respiratory failure, fever of 38.7⁰C, lactic acidosis of 4.0 mmol/L, and a mild leukocytosis of 10,890 cells/mm3 but with 10% bandemia. Spot electroencephalogram was negative for seizure activity. At this point, his antibiotics were changed to piperacillin-tazobactam and his vitals stabilized. The patient’s mental status was felt to have improved as the patient was now following commands, but he now had left arm weakness and continued aphasia. Repeat CT of the head without contrast was performed, now 17 days after fever onset, which showed worsening hypoattenuation in the left temporal lobe as well as a new right temporal lobe hypoattenuation, which was interpreted by the radiologist to be concerning for worsening edema and infarct (Figure 2).

**Figure 3:**
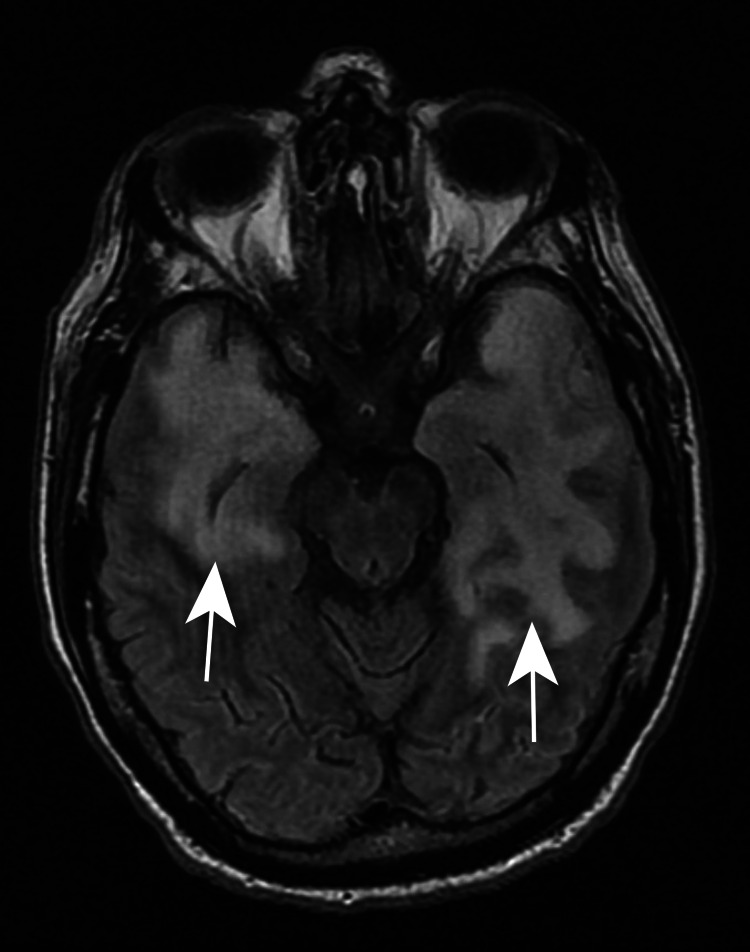
MRI of the head with extensive confluent vasogenic edema within the bitemporal lobes

Lumbar puncture was performed and cerebrospinal fluid analysis showed WBC: 14 cells/mm3 (84% lymphocytes, 16% monocytes, 0% granulocytes), RBC: 106 cells/mm3, protein: >600mg/dL, glucose: 41 mg/dL. MRI of the head was noted to have extensive confluent vasogenic edema within the bitemporal lobes with involvement of the subcortical U-fibers and scattered areas of leptomeningeal enhancement and hemorrhage (Figure 3).

HSV-1 polymerase chain reaction (PCR) on CSF was positive, which confirmed the diagnosis of HSV-1 encephalitis.

After the repeat CT head showed bilateral temporal lobe lesions, he was empirically started on IV acyclovir, and infectious diseases was consulted. After confirmation of HSV encephalitis by CSF analysis, the patient was treated with 21 days of IV acyclovir 10mg/kg every eight hours.

One week after completion of the acyclovir course, the patient’s mental status was not at baseline and he had a repeat lumbar puncture. HSV-1 PCR was still positive but there was improvement in CSF studies (WBC 13 cells/mm3, RBC 25 cells/mm3, protein 480 mg/dL, glucose 104 mg/dL). He was restarted on high dose IV acyclovir 20mg/kg every eight hours and a quantitative HSV-1 PCR was sent and found to be <100 copies. By this time he had completed an additional 21 days of IV acyclovir and therapy was stopped. At the time of completion of the second course of acyclovir, his mental status had improved from when he was diagnosed with HSV encephalitis but was not back to normal.

## Discussion

Our patient’s symptoms started at least 21 days prior to initiation of acyclovir, although he was hospitalized throughout the entire episode. The delay in diagnosis was multifactorial including his protracted clinical course, inability to obtain a healthcare surrogate for further imaging and procedures, presentation occurring while already in the hospital, and alternative diagnoses of bacterial infections. Admission was initially social and associated with his living conditions along with the inability to care for himself. His first fevers and clinical deterioration were attributed to a secondary bacterial infection and his clinical picture fluctuated over time. His symptoms and laboratory abnormalities would improve but then he would decompensate again. His mental status also fluctuated during his hospitalization. He continued to have febrile episodes, but these too would abate for a period of time without antiviral treatment. His symptoms did not fit the typical picture of HSV encephalitis, where the encephalitis is expected to worsen rapidly unless treated. In retrospect, his encephalopathy, aphasia, temporal lobe lesion, and continued fevers despite antibacterial therapy were signs of HSV-1 encephalitis. This more protracted course is less common but can occur and should not exclude the diagnosis of HSV encephalitis [2,5,6]. HSV-1 encephalitis can also be mild or biphasic with an initial deterioration followed by subsequent improvement and a second deterioration [3,6]. Thus a high index of suspicion is necessary for diagnosing HSV-1 encephalitis and it should be considered for any hospitalized patient with unresolved encephalopathy, especially with intermittent fever, temporal lobe lesions, and aphasia.

Currently, consensus is lacking regarding if the manifestation of the encephalitis is a reactivation or a primary infection, as there is evidence available to support both theories [1]. The incubation period for HSV-1 can range from two to 12 days, although not all primary infections produce symptoms [7]. Our patient had been hospitalized for more than a month prior to the presentation of encephalitis symptoms. Although he left against medical advice twice (for around 24 hours each time), he had been continuously in the hospital for the 10 days prior to symptoms. Since we were unable to obtain a social history from the patient about his activities due to his encephalopathy, it is difficult to unequivocally state if this was a primary or secondary infection. However, his time course is more consistent with the reactivation of a latent infection rather than a primary infection. For this reason, the diagnosis of HSV-1 encephalitis should remain on the differential in patients who are in the hospital prior to the onset of symptoms.

The progression of a unilateral to bilateral temporal lobe disease seen on CT head over his hospital course prior to treatment also suggests a spread from one temporal lobe to the other. Although the mechanism of entry into the CNS is disputed, the most convincing route is through the olfactory or trigeminal nerves [1,8]. After entry to the CNS, the spread between hemispheres is thought to occur via the anterior commissure [1,9]. Interestingly, the spread of his disease occurred while the patient’s mental status was stable. This could represent a delay in radiological findings of the disease or that the clinical picture may not directly equate to the severity of the imaging findings.

The patient’s lumbar puncture was repeated as he remained encephalopathic despite completion of 21 days of acyclovir treatment, and PCR revealed persistent HSV-1 DNA in the CSF 30 days after his initial lumbar puncture. Studies have shown that treatment with acyclovir leads to a decrease in the number of HSV PCR positive CSF studies over time, with 47% remaining positive after eight to 14 days of treatment, and rarely is HSV DNA isolated after 30 days [8]. Infectious Disease Society of America (IDSA) encephalitis guidelines note that patients with a negative HSV-1 CSF PCR at the end of therapy have better outcomes [10]. IDSA recommends repeat CSF testing if patients have not had the appropriate clinical response and to continue treatment if the CSF remains positive [10]. However, the recommendations do not explicitly state how long to continue the treatment course if repeat HSV-1 PCR is positive or if to adjust the acyclovir dose. Alternatively, the British Infection Association viral encephalitis guidelines recommend obtaining repeat HSV-1 CSF PCR after two to three weeks of IV acyclovir and continuing treatment and repeating weekly PCR until negative [11]. The lower relapse rates in neonates seen with high dose acyclovir 20mg/kg every eight hours have not been described with adults [12,13]. However, in adults, evaluation of acyclovir dosing was with respect to the initial treatment of HSV-1 encephalitis, not persistent detection of virus in CSF [14]. With the aforementioned information, the decision was made to reinitiate IV acyclovir for another course at a higher dose since he previously had tolerated the medication well. Quantitative HSV PCR studies have not been well evaluated but with the result of <100 copies, we felt that resistance, which is rare in non-immunosuppressed patients, was highly unlikely [8]. Delayed diagnosis and treatment and cognitive impairment prior to starting treatment also contributed to the patient’s slow recovery as more severe neurological impairment and increased time to treatment are associated with increased morbidity and mortality [8]. The patient completed an additional 21 days of acyclovir with further improvement in mental status. However, his cognitive abilities never returned to baseline.

## Conclusions

In conclusion, HSV-1 encephalitis does not always present with continued rapid progression of the disease and a patient’s presentation may remain stable without treatment for weeks. Patients that are hospitalized are still at risk of HSV-1 encephalitis as reactivation of latent virus is likely one of the prominent mechanisms of disease initiation. The diagnosis of HSV-1 encephalitis should be considered in all patients with continued encephalopathy and imaging findings of a temporal lobe lesion, especially if aphasia and fever are present. Finally, repeat CSF testing for HSV-1 should be performed in patients without an appropriate clinical response despite treatment and antiviral therapy should be continued if PCR remains positive.

## References

[1] Bradshaw MJ, Venkatesan A (2016). Herpes simplex virus-1 encephalitis in adults: pathophysiology, diagnosis, and management. Neurotherapeutics.

[2] Croll BJ, Dillon ZM, Weaver KR, Greenberg MR (2017). Subtle presentation of herpes simplex encephalitis. Am J Emerg Med.

[3] Kennedy PG (2004). Viral encephalitis: causes, differential diagnosis, and management. J Neurol Neurosurg Psychiatry.

[4] Whitley RJ (2006). Herpes simplex encephalitis: adolescents and adults. Antiviral Res.

[5] Johnson RT (1996). Acute encephalitis. Clin Infect Dis.

[6] Vachalová I, Kyavar L, Heckmann JG (2013). Pitfalls associated with the diagnosis of herpes simplex encephalitis. J Neurosci Rural Pract.

[7] Kimberlin DW, Rouse DJ (2004). Clinical practice. Genital herpes. N Engl J Med.

[8] Tyler KL (2004). Herpes simplex virus infections of the central nervous system: encephalitis and meningitis, including Mollaret's. Herpes.

[9] Jennische E, Eriksson CE, Lange S, Trybala E, Bergström T (2015). The anterior commissure is a pathway for contralateral spread of herpes simplex virus type 1 after olfactory tract infection. J Neurovirol.

[10] Tunkel AR, Glaser CA, Bloch KC (2008). The management of encephalitis: clinical practice guidelines by the Infectious Diseases Society of America. Clin Infect Dis.

[11] Solomon T, Michael BD, Smith PE (2012). Management of suspected viral encephalitis in adults--Association of British Neurologists and British Infection Association National Guidelines. J Infect.

[12] Whitley R (2004). Neonatal herpes simplex virus infection. Curr Opin Infect Dis.

[13] Skelly MJ, Burger AA, Adekola O (2012). Herpes simplex virus-1 encephalitis: a review of current disease management with three case reports. Antivir Chem Chemother.

[14] Stahl JP, Mailles A, De Broucker T (2012). Herpes simplex encephalitis and management of acyclovir in encephalitis patients in France. Epidemiol Infect.

